# SREBP-1 Transcription Factors Regulate Skeletal Muscle Cell Size by Controlling Protein Synthesis through Myogenic Regulatory Factors

**DOI:** 10.1371/journal.pone.0050878

**Published:** 2012-11-30

**Authors:** Kevin Dessalle, Vanessa Euthine, Stéphanie Chanon, Joffrey Delarichaudy, Isao Fujii, Sophie Rome, Hubert Vidal, Georges Nemoz, Chantal Simon, Etienne Lefai

**Affiliations:** 1 CarMeN Laboratory, INSERM U1060, INRA 1235, University Lyon1, Oullins, France; 2 Laboratory of Clinical Pharmacology and Therapeutics, Faculty of Pharmaceutical Sciences, Sojo University, Kumamoto-city, Japan; University of Minnesota Medical School, United States of America

## Abstract

SREBP-1 are ubiquitously expressed transcription factors, strongly expressed in lipogenic tissues where they regulate several metabolic processes like fatty acid synthesis. In skeletal muscle, SREBP-1 proteins regulate the expression of hundreds of genes, and we previously showed that their overexpression induced muscle atrophy together with a combined lack of expression of myogenic regulatory factors. Here we present evidences that SREBP-1 regulate muscle protein synthesis through the downregulation of the expression of MYOD1, MYOG and MEF2C factors. In myotubes overexpressing SREBP-1, restoring the expression of myogenic factors prevented atrophy and rescued protein synthesis, without affecting SREBP-1 action on atrogenes and proteolysis. Our results point out the roles of MRFs in the maintenance of the protein content and cell size in adult muscle fibre, and contribute to decipher the mechanisms by which SREBP-1 regulate muscle mass.

## Introduction

Loss of skeletal muscle mass occurs during aging (sarcopenia), disease (cachexia), or inactivity (atrophy) and results from an imbalance between the rate of muscle protein synthesis and degradation [1]. In a previous study, we identified SREBP-1 transcription factors as regulators of muscle mass, showing that increasing SREBP-1 nuclear content induces both in vitro and in vivo muscle cell atrophy [2]. The Sterol Regulatory Element Binding Proteins (SREBP) transcription factors belong to the basic helix-loop-helix leucine zipper family of DNA binding proteins [3]. The three isoforms are encoded by two distinct genes, *Srebf1* and *Srebf2,* and vary in structure, regulation, and functions: SREBP-1a and SREBP-1c proteins are key actors of the regulation of genes related to lipid metabolism, whereas SREBP-2 has been more closely associated to cholesterol synthesis and accumulation [4].

Beyond their strong expression in tissues with high lipogenic capacities like liver and adipose tissues, the SREBP-1 proteins are ubiquitous transcription factors with significant expression in muscle cells [5], [6]. In muscle, SREBP-1 expression is induced by activation of the PI3K/Akt and the MAP kinase pathways by insulin and growth factors [6]–[10], suggesting additional functions of these transcription factors in a tissue with a low rate of lipid synthesis. Several studies have been performed to identify SREBP-1 target genes using microarray analysis [11]–[14] and to characterize the role of SREBP-1a and -1c in skeletal muscle [2], [15], [16].

In SREBP-1 overexpressing cells, we have previously observed a combined decrease in the expression of the myogenic regulatory factors (MRFs) MYOD1, MYOG and MEF2C [2]. The MRFs are muscle-specific transcription factors that induce the expression of muscle-specific genes during the myogenic differentiation process [17], [18]. They are also still expressed in adult fibres and differentially accumulate in the various fibre types [19], [20]. MRFs may be required to maintain muscle homeostasis, and may play a role in muscle plasticity in response to both hypertrophic (ie exercise) and atrophic (ie denervation) stimuli [21]–[23].

We explored here the molecular mechanisms by which SREBP-1 proteins affect the size of differentiated myotubes, and the involvement of MRFs in this process. We investigated in particular the role of MRFs in the regulation of protein synthesis, and in the expression of ubiquitin ligases that control muscle protein degradation.

## Materials and Methods

### Culture of Skeletal Muscle Cells

For human primary myotubes, muscle biopsies were taken from healthy lean subjects who participated in a global study on insulin action on gene expression. All participants gave their written consent after being informed of the nature, purpose and possible risks of the study. The experimental protocol (‘Clamp-Gene Study’, agreement number 2003-039/125A) was approved by the Ethical Committees of the Hospices Civils de Lyon and performed according to the French legislation (Huriet law). Muscle biopsies were taken from the vastus lateralis muscle under local anaesthesia and in a fasted condition. The myoblasts were purified and differentiated myotubes were prepared according to the procedure previously described in detail [24].

### Fusion Index

Cultures were fixed and myotubes were immunostained using anti-myosin antibody [25]. Nuclei were labelled with DAPI using Vectashield mounting medium for fluorescence (Vector, USA). Nuclei were counted at least in eight randomly chosen microscope fields (2 culture dishes for each of the experimental condition, 4 fields in each dish) at a magnification of x100. One microscope field usually contained between 200 and 400 nuclei. The fusion index is defined as the number of nuclei in myotubes divided by the total number of nuclei.

### Expression Vectors and Generation of Recombinant Adenoviruses

Construction of expression vectors encoding mature nuclear forms of human SREBP-1a and SREBP-1c was described previously. Recombinant adenoviruses expressing MYOG or MEF2C were generated using the same procedure [7]. Recombinant adenovirus expressing the human MYOD1 protein is a generous gift from Dr Teruhisa Miike (Department of Child Development, Kunamoto University, Japan) [26].

### Inhibition of SREBP-1 Expression in Human Muscle Cells

Inhibition of SREBP-1 expression was performed by RNA interference. SiRNA against SREBP-1 (SI02662877) and control (Allstars negControl) were from Qiagen (Courtaboeuf, France). Myotubes were transfected for 48 h with siRNA at 20 nM final using the Hiperfect transfection reagent (Qiagen) according to the manufacturer’s protocol.

### Measurement of Protein Synthesis

Myotubes were infected for 48 h with adenovirus overexpressing GFP, SREBP-1a or SREBP-1c with or without MYOD1, MEF2C or MYOG, and protein synthesis rates were assayed as described [25]. Briefly, culture medium was then replaced by experimental medium containing 2 µCi/mL of [^3^H]-L-Tyrosine and non-radioactive tyrosine up to 2 mM, for 2 hours. Culture monolayer was washed five times with ice-cold PBS. Cells were scraped in lysis buffer, an aliquote was taken for Bradford protein analysis and the rest of protein was precipitated with 10% trichloroacetic acid (TCA). Samples were incubated at least 1 hour at 4°C and then centrifuged at 12,000 g for 10 min to separate pellet containing labelled neosynthesized proteins from supernatant, containing the pool of non-incorporated [^3^H]-L-Tyrosine. Pellet was then dissolved in basic buffer. Determination of radioactivity was performed in a Packard liquid-scintillation spectrometer.

### Measurement of Protein Degradation

Rates of protein degradation were assayed according to [25], [27] by monitoring the release of TCA-soluble radioactivity in the culture medium at defined time. Proteins were radiolabelled by incubating the cells with 2 µCi/mL of [^3^H]-L-Tyrosine in differentiating conditions for 2 days. Human myotubes were then infected with adenovirus overexpressing GFP, SREBP-1a or SREBP-1c for 48 h. Proteolysis was evaluated as the percentage of TCA-soluble radioactivity released in the medium reported to total incorporated radioactivity.

### Protein Expression Analysis by Immunocytofluorescence

Measurement of the area of immunofluorescence-labelled myotubes was performed as previously fully described [25].

### Protein Expression Analysis by Western Blotting

Cells were harvested, extracted and immunoblotted as previously described [7]. The total cell lysates were immunoblotted with the following antibodies: anti-FoxO1 (#05-1075), anti-phospho(Ser256)FoxO1 (#9461), anti-phospho(Ser253)FoxO3a (#9466) and anti-MEF2C (#5030) were from Cell Signaling Technology (Beverly, MA, USA); anti-FOXO3a (#04-1007) from Millipore (Billerica, MA, USA); anti-MYOD1 (sc760), anti-MYOG (sc576) and anti-TroponinI-SS (sc8119) from Santa Cruz Biotechnology (Santa Cruz, CA, USA); anti-MURF1 (ab77577) from Abcam Biotechnology (Cambridge, UK) ; anti- alpha-tubulin (T5168) from Sigma (L’Isle-d’abeau, France); and anti-sarcomeric MHC (MF-20) were from the Developmental Studies Hybridoma Bank (Iowa City, IO, USA).

### Luciferase Assay

The MuRF-1 reporter plasmid was constructed by ligating PCR fragments of the human genomic region upstream of the transcription start site of the MuRF1 gene into the pGL3-Enhancer reporter (Promega). The MuRF1 genomic fragment was generated by PCR (primers upon request at lefai@univ-lyon1.fr) to obtain the construction −1337/+90 (according to the transcription starting site). Next, deletions were performed on the −1337/+90 construct to obtain the −540/+90 and −237/+90 constructs.

Cell transfection and luciferase assays were performed as previously described [7].

### Quantification of mRNAs by Real-time RT-PCR

Total RNA was isolated and first-strand cDNAs were synthesized as previously described [7]. A list of the primers and real-time PCR assay conditions are available upon request (lefai@univ-lyon1.fr). The results were normalized using RPLP0 mRNA concentration, measured as reference gene in each sample.

## Results

### Overexpression of SREBP-1 in Muscle Cells Reduces Protein Synthesis

**Figure 1:**
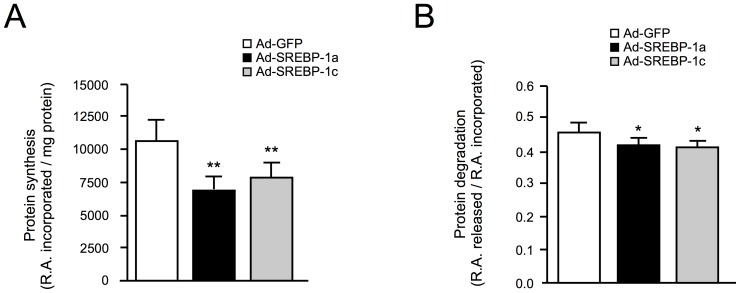
SREBP1s decrease protein synthesis and degradation in human primary myotubes. Protein synthesis and degradation rates were determined for 48 hours after infection of human primary myotubes with recombinant adenoviruses encoding GFP (white), SREBP-1a (black) or SREBP-1c (grey). (A) The rate of protein synthesis was measured by adding [^3^H]-tyrosine to culture medium, and counting the radioactivity (RA) present in trichloroacetic acid (TCA) precipitates proteins of the cells, reported to the total protein content. (B) Myotubes were labelled with [^3^H]-tyrosine for 48 h prior to adenoviruses infections. The release of radioactivity (RA) in the TCA soluble fraction was measured and expressed as a ratio to total incorporated RA. The results are the means ± SE of 8 determinations. **: different from control, p<0.01, *: different from control, p<0.05.

To decipher the molecular mechanisms by which SREBP-1 overexpression induces atrophy in muscle cells, we first examined the impact of SREBP-1a and -1c overexpression on protein synthesis and degradation rates (Figure 1). In human primary differentiated myotubes, overexpression of both SREBP-1a and SREBP-1c significantly decreased protein synthesis rates (−33%, p = 0,002 and −26%, p = 0,003 respectively), compared to GFP overexpression (Figure 1A). Under the same conditions, a slight decrease in protein degradation rates was also observed, (−8% and −10% respectively) (Figure 1B). The amplitude of changes in protein synthesis and degradation we observed thus indicates that SREBP-1 induced atrophy is due to an inhibition of muscle protein synthesis.

### Forced Expression of MRFs Reverses the SREBP-1 Effects on Muscle Cells

**Figure 2:**
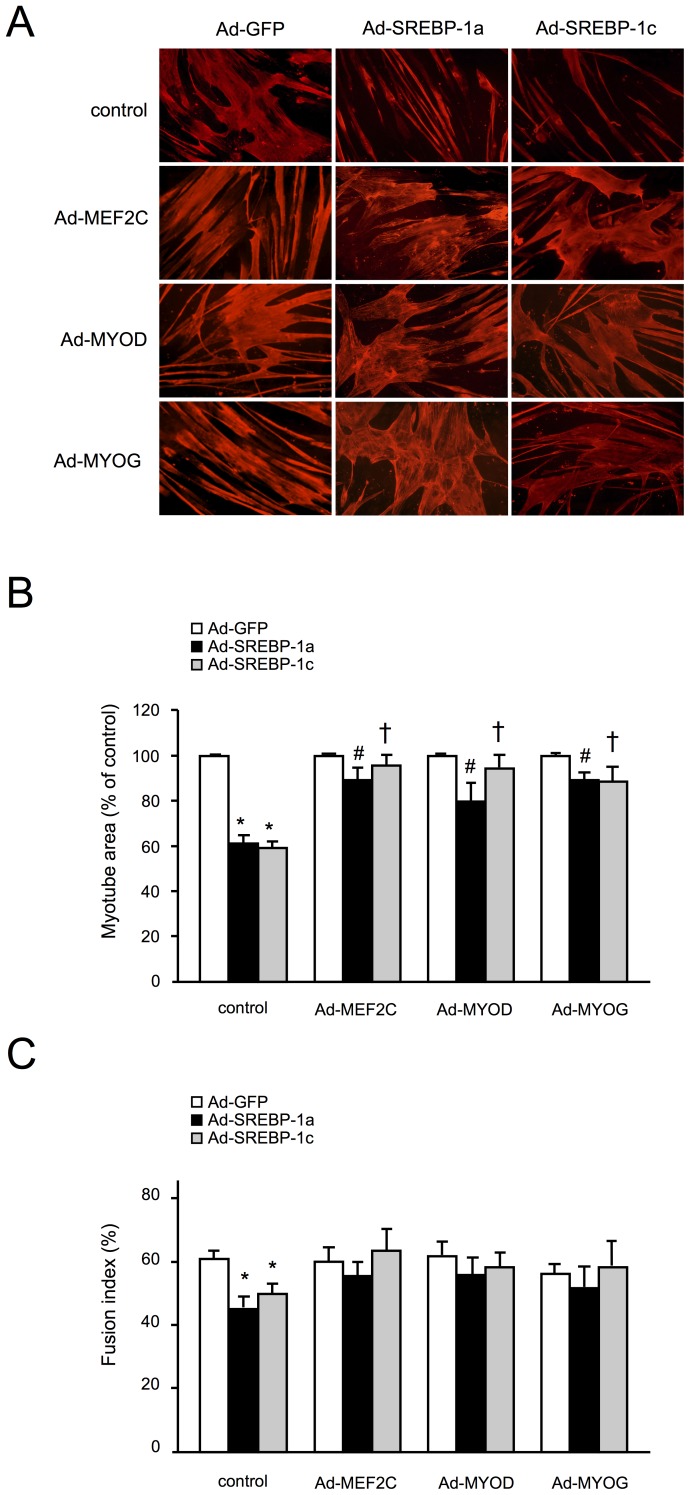
Expression of MEF2C, MYOD1 and MYOG inhibits SREBP-1 induced atrophy in myotubes. Human myotubes were infected for 48 h with recombinant adenoviruses encoding GFP, SREBP-1a or SREBP-1c, in combination with viruses expressing MEF2C, MYOD1, MYOG or GFP (control). (A) Representative images of myotube immunostaining with Myosin Heavy Chain antibody. (B) Myotube areas were measured as cell surface determination from MHC immunostaining pictures. The results are the means ± SE of 5 independent experiments (with 10 fields considered for each condition). *p<0.001 compared to Ad-GFP infected cells. #p<0.05 compared with Ad-SREBP-1a alone ; †p<0.05 compared with Ad-SREBP-1c alone; (C) Fusion index. The number of nuclei (total and in myotubes) was counted in all 12 conditions (with at least 4 fields for each condition). The results are the means ± SE of 3 independent experiments. *p<0.05 compared to Ad-GFP infected cells.

We previously demonstrated that SREBP-1 overexpression inhibits the expression of several MRFs through an induction of both BHLHB2 and BHLHB3 transcriptional repressors [2]. We examined here if forced expression of these MRFs in SREBP-1 overexpressing myotubes could reverse the induced atrophy. As shown in Figure 2, immunostaining of myotubes reveals that MEF2C, MYOD1 and MYOG, when co-expressed with either SREBP-1a or -1c, restored almost completely the expression of sarcomeric myosin heavy chain protein (Figure 2A). In all cases, when lowered MRFs expression was compensated for by viral expression, the SREBP-1 induced atrophy was limited as shown by the preservation of myotube area (Figure 2B).

We next quantified the fusion index (ratio of the number of nuclei in myotubes to the total number of nuclei) in all the conditions where SREBP-1 and/or MRFs were overexpressed (Figure 2C). We first observed that the overexpression of either SREBP-1a or -1c induced a significant decrease in fusion index (Figure 2C, control). A 15% loss of nuclei in SREBP-1 overexpressing myotubes is observed when myotube areas showed a reduction of 40% (Figure 2B). Notably, when overexpressed alone, none of the studied MRFs increased the fusion index compared to GFP (white bars), suggesting that the in vitro differentiation was complete and that the remaining myoblasts were unable to fuse with myotubes. Finally, when SREBP-1 proteins were co-expressed with MRFs, the atrophy was prevented and the decrease in fusion index was abolished (black and grey bars). Thus, the restoration of MRF expression in SREBP-1 overexpressing myotubes can prevent the atrophic process and inhibit the loss of nuclei in differentiated myotubes.

### Inhibition of SREBP-1 Expression Increases Protein Synthesis and Degradation

**Figure 3:**
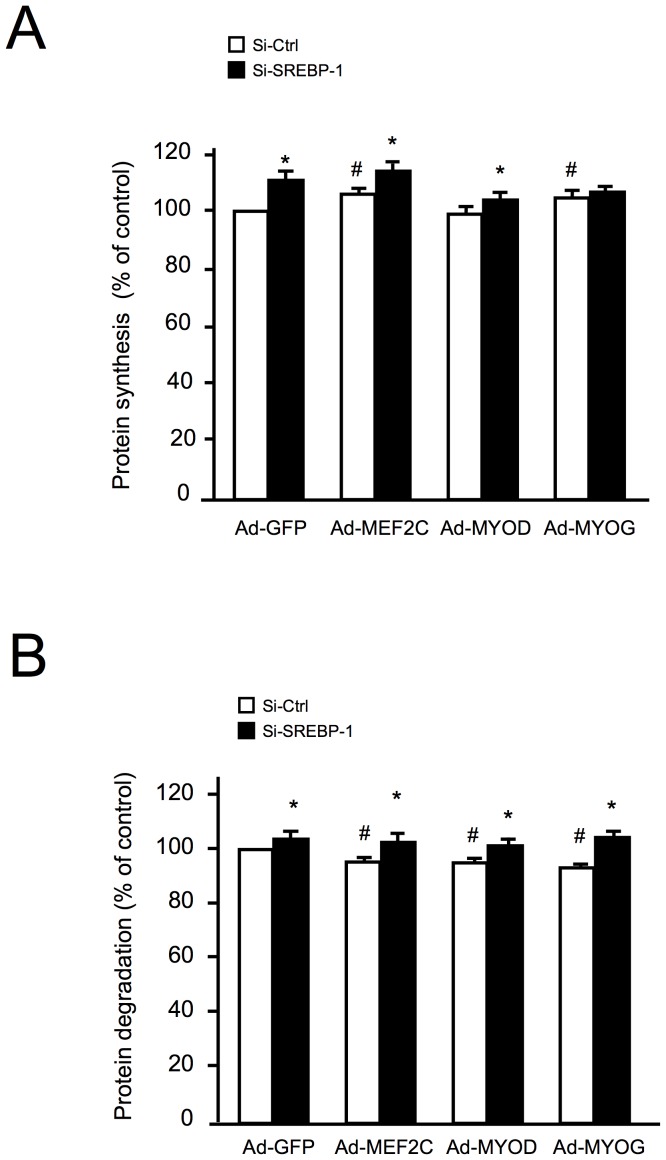
siRNA-mediated inhibition of SREBP-1 affects protein synthesis and degradation rates. Protein synthesis and degradation rates were determined in human primary myotubes under GFP or MRFs overexpression. Cells were transfected with siRNAs (control or against SREBP-1) and infected with recombinant adenovirus for 48 hours. The results are the means ± SE of 4 determinations. *p<0.05 compared to Ad-GFP infected cells. #p<0.05 compared to control siRNA.

As overexpression of SREBP-1 proteins mainly affected protein synthesis, and more mildly decreased protein degradation (Figure 1), we next studied the effects of siRNA mediated SREBP-1 depletion on protein metabolism, with or without MRF overexpression. As shown in Figure 3, compared to control conditions, overexpression of MRFs increased protein synthesis and decreased protein degradation (white bars). When the expression of SREBP-1 was inhibited, protein synthesis was higher compared to control siRNA conditions (Figure 3A). The same was observed for protein degradation (Figure 3B), with SREBP-1 extinction inducing a slight but significant increase. As the results of SREBP-1 down-regulation were opposite of those of SREBP-1 overexpression, we then conclude that changes in SREBP-1 protein content, either positive or negative, can modulate protein turnover in myotubes.

### Forced Expression of MRFs Prevents the SREBP-1s Induced Decrease in Protein Synthesis

**Figure 4:**
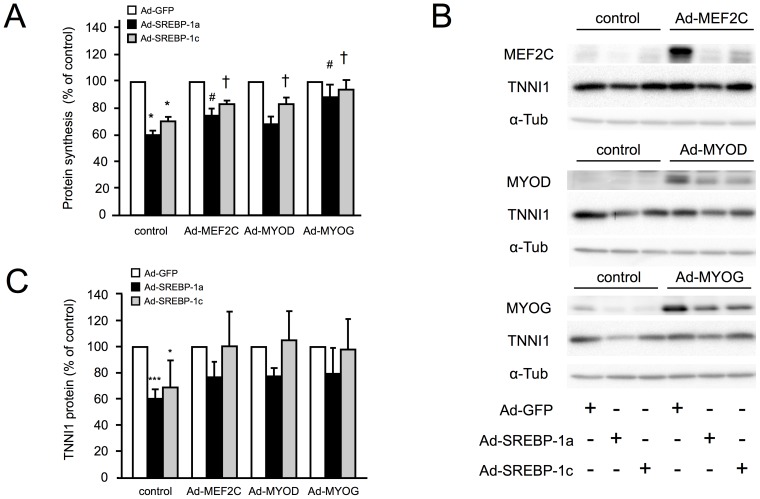
Expression of MEF2C, MYOD1 and MYOG are necessary to maintain muscle cell protein synthesis and expression of sarcomeric proteins. Human myotubes were infected for 48 h with recombinant adenoviruses encoding GFP (white), SREBP-1a (black) or SREBP-1c (grey) in combination with viruses expressing MEF2C, MYOD1, MYOG or GFP (control). (A) Protein synthesis rates were measured in all different conditions. The results are the means ± SE of 5 determinations. *p<0.001 compared to Ad-GFP infected cells. #p<0.05 compared with Ad-SREBP-1a alone ; †p<0.05 compared with Ad-SREBP-1c alone (B) Representative images of TNNI1 immuno-detection by western blotting from total cell extract of myotubes. (C) Quantification of TNNI1 content in each condition, normalized by tubulin and expressed as percentage of control. The results are the means ± SE of 3 determinations.

To explore the mechanisms by which MRFs can counteract the SREBP-1 induced reduction in cell size, we measured protein synthesis rates in conditions in which expression of each MRF is restored. As shown in Figure 4A, the dramatic decrease in protein synthesis rate induced by SREBP-1 was partly abolished when the expression of MEF2C, MYOD1 or MYOG was maintained. The absence of decrease in protein synthesis rate was particularly evident when MYOG expression was maintained, with no differences in protein synthesis whether SREBP-1a, SREBP-1c or GFP were overexpressed.

To evaluate the impact of MRFs on sarcomeric protein content, we next quantified protein levels of Troponin I slow-twitch isoform (TNNI1), in different conditions. As observed for MHC expression (Figure 2A), TNNI1 expression quantified by western blotting was significantly decreased when SREBP-1 were expressed alone, but the decrease was reduced and non significant when one of the MRFs was co-expressed with SREBP-1 (Figures 4B and 4C). We thus conclude that the decrease in protein synthesis rate in atrophic muscle cells overexpressing SREBP-1 is mainly due to the inhibition of the expression of MRFs, and that maintenance of MRFs can protect muscle specific gene expression from the inhibition induced by SREBP-1 increase.

### MRF-independent Regulation of Atrogenes Expression by SREBP-1

We previously observed that SREBP-1 overexpression led to opposite regulation of FBXO32 (Atrogin-1) and TRIM63 (MuRF1), the two E3-ubiquitin ligases involved in muscle protein degradation through the Ubiquitin Proteasome System (UPS) [2].

**Figure 5:**
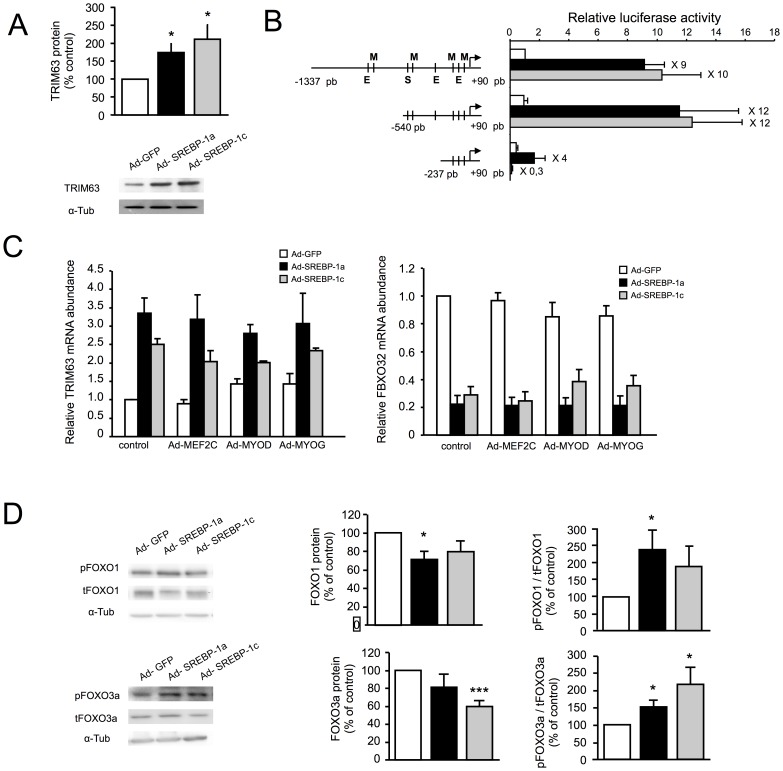
Opposite and indirect regulations of atrogenes by SREBP-1. Human myotubes were infected for 48 h with recombinant adenoviruses encoding GFP (white), SREBP-1a (black) or SREBP-1c (grey). (A) Protein levels of TRIM63 in myotubes overexpressing GFP, SREBP-1a or SREBP-1c. An illustrative immunoblot and quantification of the results are shown. The protein level of alpha-tubulin was used for normalization. The results presented are the means ± SE of 4 determinations. *p<0,05; **p<0.001 (B) TRIM63 proximal promoter activity on human myotubes. S represent Sterol Regulatory Element putative motif, E represent E-box motifs and M represent MRFs motifs. The results presented are the means of 5 determinations. (C) mRNA levels of atrogenic factors (FBXO32 and TRIM63) in response to GFP, SREBP-1a or SREBP-1c overexpression in combination with adenvirus-mediated overexpression of MEF2C, MYOD1, MYOG or GFP (control). The results presented are the means ± SE of 3 determinations. (D) Protein levels of tFOXO1 (total) and pFOXO1 (Ser256 phosphorylated) and tFOXO3 (total) and pFOXO3 (Ser253 phosphorylated) were determined by western blot experiments in myotubes overexpressing GFP, SREBP-1a or SREBP-1c. Immunodetection of alpha-tubulin was performed to normalize for the amount of loaded protein. Illustrative immunoblots are shown on the left, and the results of quantification are shown on the right. The results are presented as mean ± SE of 6 separate experiments. *p<0,05; **p<0.001.

In SREBP-1 overexpressing myotubes, TRIM63 mRNA expression is enhanced in conditions in which MRFs expression is abolished [2], and we verified here that the protein levels were also increased (Figure 5A). To confirm this MRF-independent activation of TRIM63 by SREBP-1, we performed luciferase assays to measure the human TRIM63 proximal promoter activities (Figure 5B). We identified a 303 bp region (−540 to −237) as the main region mediating the effects of SREBP-1s. This region contains a Sterol Regulatory Element putative motif [28] and E-boxes. In addition, the proximal promoter contains several MRF motifs, suggesting a possible direct positive regulation by MRFs. However, re-expression of MRFs did not modify TRIM63 expression in SREBP-1 overexpressing myotubes (Figure 5C), showing that the promoter is not controlled by MRFs, and might instead be directly activated by SREBP-1.

By contrast, SREBP-1 overexpression induced a decrease in FBXO32 expression [2]. As MYOG is known to bind atrogene promoters and activate atrogene expression [29], [30], we measured mRNA levels of FBXO32 when MRFs were re-expressed in the presence of overexpressed SREBP-1. As shown in Figure 5C (right panel), none of them could restore FBXO32 expression in SREBP-1 induced atrophy. We next examined the expression and phosphorylation status of FOXO1 and FOXO3 transcription factors, which are known to be major transcriptional regulators of FBXO32 expression. As shown in Figure 5D, both SREBP-1a and -1c induced a decrease in the total amount of FOXO proteins together with a significant increase in their phosphorylation. Under SREBP-1 overexpression, independently of MRFs expression, FOXO proteins are thus excluded from the nucleus, which likely explains the observed decrease in FBXO32 expression.

## Discussion

SREBP-1 transcription factors are known to regulate expression of hundreds of genes in liver [12], [13], fibroblasts [11] and muscle cells [15]. In muscle, we previously reported that their overexpression leads to atrophy of both differentiated myotubes in vitro, and tibialis muscle in vivo, via the induction of BHLHB2/B3 transcriptional repressors, and that the expression of MRFs was drastically reduced in these conditions [2]. MRFs are still expressed in differentiated myotubes [21], [31], and participate in the expression of sarcomeric proteins [32]. MYOG is involved in the maintenance of the neuromuscular junction [30], [33]. It has also been shown that inhibiting MYOD1 degradation can maintain the muscle mass in conditions of FBX032 accumulation [34]. In the present work, we determined the impact of SREBP-1 overexpression on protein metabolism in differentiated myotubes, and we investigated the role of MRF repression in the atrophic response, as evaluated by the reduction in cell size and by the loss of muscle-specific proteins.

In our SREBP-1 induced atrophy model, we observed that the reduction in myotube size could be attributed to a marked decrease in protein synthesis not compensated for by a small decrease in proteolysis. Conversely, the inhibition of SREBP-1 expression affected protein synthesis and degradation rates in an opposite manner, indicating that both an increase and a decrease in SREBP-1 nuclear content modulate muscle protein content.

The SREBP-1 induced atrophy was also characterized by a loss of myonuclei that accompanied the decrease in protein content. The loss of nuclei (mainly by apoptosis) during atrophic process has been extensively documented, even if the opposite (i.e. conservation of nuclei number) is also described in vivo (recently discussed in [35]). In our model, whether the number of nuclei in myotubes is directly controlled by MRFs, or whether the loss is a consequence of the dramatic decrease in cytoplasm volume remains to be determined. Nevertheless, when MRF expression was maintained in SREBP-1 overexpressing myotubes, the loss of myonuclei was prevented, excluding a direct effect of SREBP-1 on the number of nuclei per myotube. The re-expression of each of the considered MRF was also able to partially restore overall protein synthesis, and to prevent the loss of cell content in the muscle specific proteins MHC and Troponin I, showing that the combined decrease in MRFs expression in differentiated myotubes was responsible for protein synthesis inhibition.

SREBP-1 proteins also tended to decrease protein degradation rate, but examination of the atrogenes TRIM63 and FBXO32 showed that SREBP-1 promoted a dramatic increase in TRIM63 expression together with a decrease in FBXO32. These changes in expression remained when MRFs expression was restored and the atrophy was prevented. We can then exclude the participation of MRFs in the SREBP-1 regulation of atrogenes. We conclude that the main factor inducing myotube atrophy under SREBP-1 overexpression is the inhibition of MRF expression by the SREBP-1/BHLHB2/3 pathway, and that MRF expression in differentiated myotubes is necessary for the maintenance of mature fibre size and protein content.

The control of the amount of SREBP-1 proteins in the nucleus involves regulation at several levels, including SREBP-1 gene expression, proteolytic cleavage in the endoplasmic reticulum, nuclear import and activation/degradation within the nucleus (for review see [4]). It has been recently demonstrated in liver that SREBP-1 expression is enhanced through the PKB/mTOR pathway [36], [37], and repressed through the AMPK pathway [38]. SREBP-1 is also a target of the deacetylase SIRT1, leading to an inhibition of its transcriptional activity [39], [40]. Whether these regulations have the same relevance in muscle tissue remains to be elucidated, but SREBP-1 could thus integrate signalling from nutrient sensing, energetic status and metabolic requirements towards muscle mass regulation, in pathways involving MRFs.

It will therefore be of particular interest to further study these transcription factors in pathological situations inducing muscle wasting, and also to evaluate their impact on muscle tissue in metabolic diseases where abnormalities in SREBP-1 have already been reported, such as insulin-resistance and type 2 diabetes [5], [41], [42].
